# Short term optical defocus perturbs normal developmental shifts in retina/RPE protein abundance

**DOI:** 10.1186/s12861-018-0177-1

**Published:** 2018-08-29

**Authors:** Nina Riddell, Pierre Faou, Sheila G. Crewther

**Affiliations:** 1 Department of Psychology and Counselling, School of Psychology and Public Health, La Trobe University, Plenty Rd., Bundoora, Melbourne, VIC 3083 Australia; 2 Department of Biochemistry and Genetics, La Trobe Institute for Molecular Sciences, La Trobe University, Melbourne, VIC Australia

**Keywords:** Myopia, Hyperopia, Chick, Proteomics, Mass spectrometry, Development, Emmetropization, Neurotransmission

## Abstract

**Background:**

Myopia (short-sightedness) affects approximately 1.4 billion people worldwide, and prevalence is increasing. Animal models induced by defocusing lenses show striking similarity with human myopia in terms of morphology and the implicated genetic pathways. Less is known about proteome changes in animals. Thus, the present study aimed to improve understanding of protein pathway responses to lens defocus, with an emphasis on relating expression changes to no lens control development and identifying bidirectional and/or distinct pathways across myopia and hyperopia (long-sightedness) models.

**Results:**

Quantitative label-free proteomics and gene set enrichment analysis (GSEA) were used to examine protein pathway expression in the retina/RPE of chicks following 6 h and 48 h of myopia induction with − 10 dioptre (D) lenses, hyperopia induction with +10D lenses, or normal no lens rearing. Seventy-one pathways linked to cell development and neuronal maturation were differentially enriched between 6 and 48 h in no lens chicks. The majority of these normal developmental changes were disrupted by lens-wear (47 of 71 pathways), however, only 11 pathways displayed distinct expression profiles across the lens conditions. Most notably, negative lens-wear induced up-regulation of proteins involved in ATP-driven ion transport, calcium homeostasis, and GABA signalling between 6 and 48 h, while the same proteins were down-regulated over time in normally developing chicks. Glutamate and bicarbonate/chloride transporters were also down-regulated over time in normally developing chicks, and positive lens-wear inhibited this down-regulation.

**Conclusions:**

The chick retina/RPE proteome undergoes extensive pathway expression shifts during normal development. Most of these pathways are further disrupted by lens-wear. The identified expression patterns suggest close interactions between neurotransmission (as exemplified by increased GABA receptor and synaptic protein expression), cellular ion homeostasis, and associated energy resources during myopia induction. We have also provided novel evidence for changes to SLC-mediated transmembrane transport during hyperopia induction, with potential implications for signalling at the photoreceptor-bipolar synapse. These findings reflect a key role for perturbed neurotransmission and ionic homeostasis in optically-induced refractive errors, and are predicted by our Retinal Ion Driven Efflux (RIDE) model.

**Electronic supplementary material:**

The online version of this article (10.1186/s12861-018-0177-1) contains supplementary material, which is available to authorized users.

## Background

The prevalence of myopia (short-sightedness) is increasing worldwide, and by 2050 it is expected that approximately 4.8 billion people will be myopic (almost 1 billion of whom will be highly myopic) [1]. Myopia is also a major risk factor for severe sight threatening pathologies including choroidal neovascularization, retinal detachment, glaucoma, and cataract [2–4]. Such myopia-associated pathologies are often the most frequent cause of blindness in younger age-groups e.g. [5–7], highlighting the urgent need to understand the underlying biological processes so that effective treatments can be developed.

Myopia and hyperopia (long-sightedness) occur when the eye grows too long or short, respectively, for its refractive power [8, 9]. Such a disruption in the normal emmetropization process (i.e., the active process whereby the predominantly hyperopic eyes of infants increase in size to facilitate image focus on the retina [10–14]) occurs in response to change in the visual environment early in life in all human and animal species studied to date [15–24]. Animals reared with frosted occluders or negative defocusing lenses over one eye rapidly develop axial myopia, while those reared with positive defocusing lenses exhibit slower ocular growth and refractive hyperopia [16, 24]. Myopia in humans and animal models is characterized by reduced visual acuity, axial elongation, thinning of the retina, choroid and fibrous sclera [25–36], and physiological changes in the electroretinogram [37–41]. Chick myopia develops even after optic nerve section, suggesting that ocular growth is regulated by molecular mechanisms that are predominantly local to the eye (and to the retina in particular given that it is the only light sensitive element and the first tissue in which visual processing occurs) [30, 42–46].

Exploratory gene [47–58] and protein [59–69] expression profiling has been used in extensive attempts to elucidate the retinal signalling cascades that regulate ocular growth and refractive compensation in animal models. We have recently demonstrated good concordance between the single genes and proteins implicated in these studies and those linked to myopia and its sequelae (including macular degeneration and choroidal neovascularization) in humans [56, 66, 70]. Pathway-focused analyses have begun to identify unifying themes at the transcriptome level, including increased expression of mitochondrial metabolism and inflammatory (particularly complement/coagulation) pathways in the retina/RPE/choroid during myopia induction [56–58]. Less is known about pathway expression at the protein level, as most studies to date have used low throughput gel-based methods to identify highly up- and down-regulated proteins [70]. This approach generally provides poor representation of lowly abundant proteins, highly acidic/basic proteins, and hydrophobic proteins [71]. It also precludes the analysis of whole pathways using threshold-free functional class scoring techniques like Gene Set Enrichment Analysis (GSEA). GSEA is based on the assumption that coordinated changes in the expression of genes within a pathway are likely to be biologically important, even if the changes are modest. Unlike over-representation techniques (which have been commonly used in refractive error research e.g. [51, 53, 55, 56, 72]), GSEA analyses the entire dataset without applying a cut-off. Pathway scores are based on the expression of all genes or proteins in the class. Consequently, the results are more stable and the analysis can detect coordinated but subtle changes in the expression of gene sets that are missed by single gene and over-representation analyses [73, 74].

In addition to underutilization of pathway analysis techniques, few retinal proteomic studies have compared multiple ocular growth conditions or multiple time-points [65, 66]. At the transcriptome level, studies profiling the retina during both myopia and hyperopia induction have identified many similar and distinct gene expression profiles across the two conditions [55–57]. Bidirectional responses appear comparatively rare, except when the analysis is specifically designed to identify such changes (e.g. by identifying pathways correlated with ocular refraction across myopic and hyperopic groups [57]). Studies profiling multiple time-points have demonstrated that different genes are involved in the initiation versus the progression of optically-induced refractive errors e.g. [50, 51, 57], and that some of the genes implicated during refractive error induction are also developmentally regulated (i.e., changing over time in normally developing control retina) [57].

To address the lack of high throughput retinal proteomic studies comparing multiple lenses and time-points, we have recently used LC-ESI-MS/MS to examine single differentially-expressed proteins in the retina/RPE of chicks following 6 h and 48 h of myopia induction with − 10 dioptre (D) lenses, hyperopia induction with +10D lenses, or normal no lens rearing. We identified a similar pattern of single protein changes to that reported in transcriptome studies [55–57], with significant refractive compensation and > 140 differentially abundant proteins evident in both lens groups at 6 h and 48 h. The response to negative and positive lenses was highly similar at both time-points, and only 13 proteins displayed sign-of-defocus dependent changes [66]. Many of the identified proteins had previously been linked to the development of secondary pathologies (macular degeneration and choroidal neovascularization) in humans, and proteins negatively correlated with ocular refraction across lens groups at 6 h were enriched for genes linked to human photoreceptor dystrophies and mitochondrial disorders [66]. This finding is consistent with human genetic studies showing a high incidence of refractive errors in individuals with inherited photoreceptor dystrophies [75] as well as a recent refractive error genome-wide association meta-analyses implicating ‘abnormal photoreceptor inner segment morphology’ as the most significant gene set [76]. Such changes to photoreceptor dynamics are predicted by Crewther’s Retinal Ion-Driven Fluid Efflux (RIDE) model [77].

Our earlier proteomic study [66] emphasized the relevance of the chick retina/RPE lens model to human myopia, and provided support for the early disruption of photoreceptor signalling. However, in this previous study we did not analyse changes over time within each lens condition and as such could not relate protein shifts during lens-wear to any corresponding shifts occurring in the no lens group undergoing normal emmetropization. The > 390 proteins implicated across 6 and 48 h time-points highlights the need for a pathway-based approach to adequately summarize the biological processes involved in adapting to the rapid refractive compensation and photoreceptor changes occurring during the initial hours of lens-wear. Thus, in the present study we re-analysed our previously published proteomic dataset [66] using a GSEA pathway approach with the aim of (i) improving understanding of the relationship between lens-induced pathway expression changes and normal no lens development, and (ii) identifying bidirectional and/or distinct pathway expression shifts in the two lens groups that may have potential for the development of targeted therapies to inhibit or slow the progression of excessive myopic ocular growth.

## Results

As previously reported, chicks were assigned to a lens condition (negative lens, positive lens, or no lens) on post-hatch day 5 (P5). Experimental eye refraction and axial dimensions were measured following a further 6 h (P5) and 48 h (P7) of rearing. These biometric measures (Additional file 1: Table S1) confirmed that chicks in the positive and negative lens conditions displayed compensatory changes in refraction and axial length at the 6 h time-point, and that refractive compensation was almost complete in both lens conditions by 48 h (negative lens mean refraction ± SE = − 9.70D ± 0.41D, positive lens = 7.70D ± 0.44D). Normally developing no lens chicks were approximately emmetropic at both time-points (no lens 6 h = 0D ± 0.45D, 48 h = 0.63D ± 0.16D). Statistical analyses reported in our previous publication [66] confirmed that the refractive state of all lens groups was significantly different at both time points (*p* < 0.01 for all comparisons). After biometric data collection, the retina/RPE was extracted for label-free LC-ESI-MS/MS proteomic analysis. The pre-processed LC-ESI-MS/MS matrix is provided in Additional file 2: Table S2.

The LC-ESI-MS/MS dataset was analysed using GSEA [74] to identify enriched Reactome pathways between lens groups at each time-point, and within each of the three lens groups over time (i.e., between the 6 and 48 h time-points). Eighty-three of the 431 Reactome pathways measured in the dataset were significantly enriched (FDR < 0.05) in one or more of these pairwise comparisons. To simplify the interpretation and remove redundancy from the pathway-level findings, pathways with the same leading edge subset (LES) proteins driving their enrichment were grouped into clusters (see the Methods section for further explanation of the LES). Additional file 3: Figure S1-S2 provides a visual representation of the pathway clustering.

This grouping revealed nine clusters of pathways representing common cellular processes related to nucleocytoplasmic transport, gene expression regulation, translation, mitochondrial protein import, rab GTPases, integration of energy metabolism, ion and vascular homeostasis, signal transduction, and solute transport (Table 1). As outlined in Table 1, pathways from the nucleocytoplasmic transport cluster were up-regulated over time between 6 and 48 h in normally developing no lens chicks. Pathways from the remaining 8 clusters were down-regulated over time during normal development. Up-regulation of pathways from the nucleocytoplasmic transport cluster was preserved in the positive lens condition; however, positive and negative lens-wear perturbed the timing, strength, and/or direction of all other normal developmental shifts. The following sections present the pairwise enrichment results for each cluster in detail.

**Table 1:** Summary of pathway enrichment within each cluster

Cluster name	Pathways in cluster	Significant (FDR < 0.05) pathway expression changes
Nucleocytoplasmic transport	38	24 pathways up over time in no lens & positive lens
		8 pathways up over time in no lens
		6 pathways up over time in positive lens
Regulation of gene expression	8	1 pathway down in negative & positive vs. no lens at 6 h, & down over time in no lens
		6 pathways down in positive vs. no lens at 6 h, & down over time in no lens
		1 pathway down in negative vs. no lens at 48 h
Translation	22	22 pathways down over time in no lens
Mitochondrial import	1	1 pathway down over time in no lens
rab GTPase	1	1 pathway down over time in no lens
Integration of energy metabolism	2	2 pathways down over time in no lens
Ion and vascular homeostasis	6	3 pathways up over time in negative lens, & down over time in no lens
		1 pathway down in negative vs. positive lens at 6 h & 48 h
		2 pathways down in negative vs. positive lens at 6 h
Signal transduction	3	1 pathway up over time in negative lens
		2 pathways down over time in no lens
Solute transport	2	1 pathway down over time in no lens
		1 pathway up in positive vs. no lens at 48 h

### Nucleocytoplasmic transport cluster

**Fig. 1:**
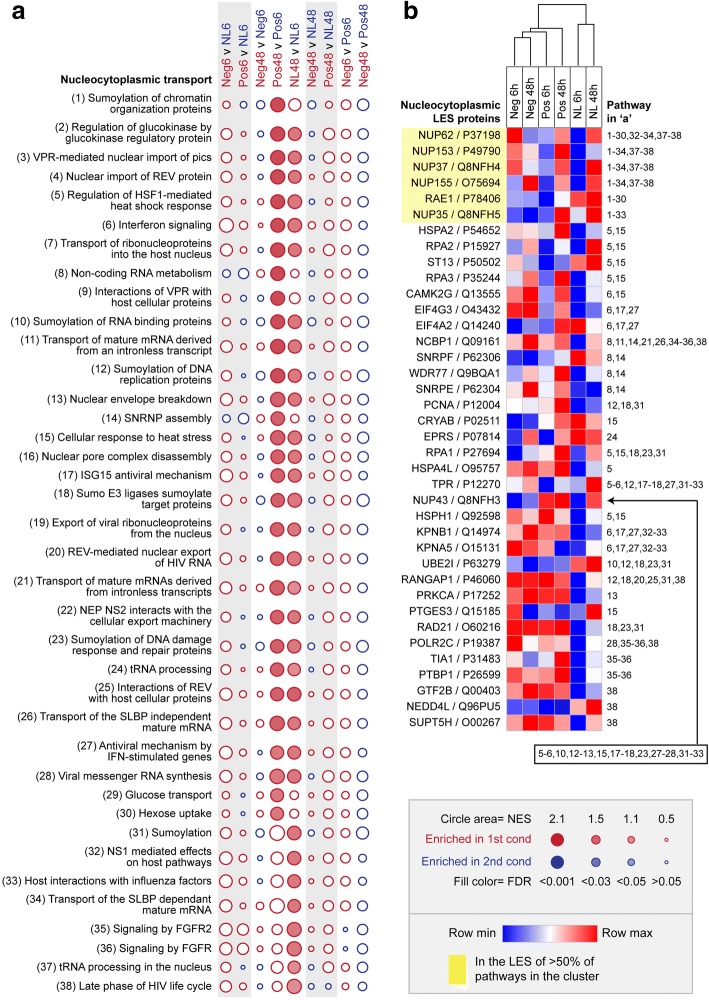
Pathway enrichment in the nucleocytoplasmic transport cluster. **a** Bubble plot illustrating the normalized enrichment score (NES) and false discovery rate (FDR) across all pairwise comparisons for the thirty-eight enriched pathways in the ‘nucleocytoplasmic transport’ cluster. Statistically significant enrichments (FDR < 0.05) are shown as filled circles. **b** Heat map showing the mean label-free quantification (LFQ) intensity across lens conditions for all leading edge subset (LES) proteins contributing to the enrichment of one or more nucleocytoplasmic pathways. Note that the LES varied across pathways that were significantly enriched over time in both positive and no lens conditions. Full details of the LES for each comparison are provided in, Additional file 4 Figure S3

Thirty-eight of the 83 implicated pathways were assigned to the ‘nucleocytoplasmic transport’ cluster on the basis of common LES proteins. The pairwise enrichment profiles for the pathways in this cluster are represented in each row of the Fig. 1a bubble plot; the lens relative to no lens comparisons at 6 and 48 h are highlighted in grey, and within group changes over time are interposed between them. Statistically significant enrichments (FDR < 0.05) are indicated by circle fill, and normalized enrichment scores (NES) are indicated by circle size. The NES is the primary statistic for comparing enrichment results; it represents the degree to which proteins from the pathway were over-represented at the top or bottom of the ranked protein list (after accounting for variations in pathway size). Accordingly, larger circle sizes indicate a greater difference in pathway expression between the two conditions.

As illustrated in Fig. 1a, the 38 pathways in the nucleocytoplasmic transport cluster were up regulated over time between 6 h and 48 h in chicks wearing positive lenses and/or no lens. Most of these pathways were related to the nucleocytoplasmic transport of RNA and proteins (including ribosomal proteins), with LES comprised primarily of nuclear pore complex subunits (NUP62, NUP153, NUP37, NUP155, NUP35; Fig. 1b). Two heat shock pathways were also implicated (‘regulation of HSF1-mediated heat shock response’ and ‘cellular response to heat stress’; Fig. 1a), with LES comprised of nuclear pore complex subunits and proteins linked to heat stress and DNA damage (including CRYAB, HSPA2, HSPH1, RPA1, RPA2, RPA3, and ST13; Fig. 1b).

### Regulation of gene expression cluster

**Fig. 2:**
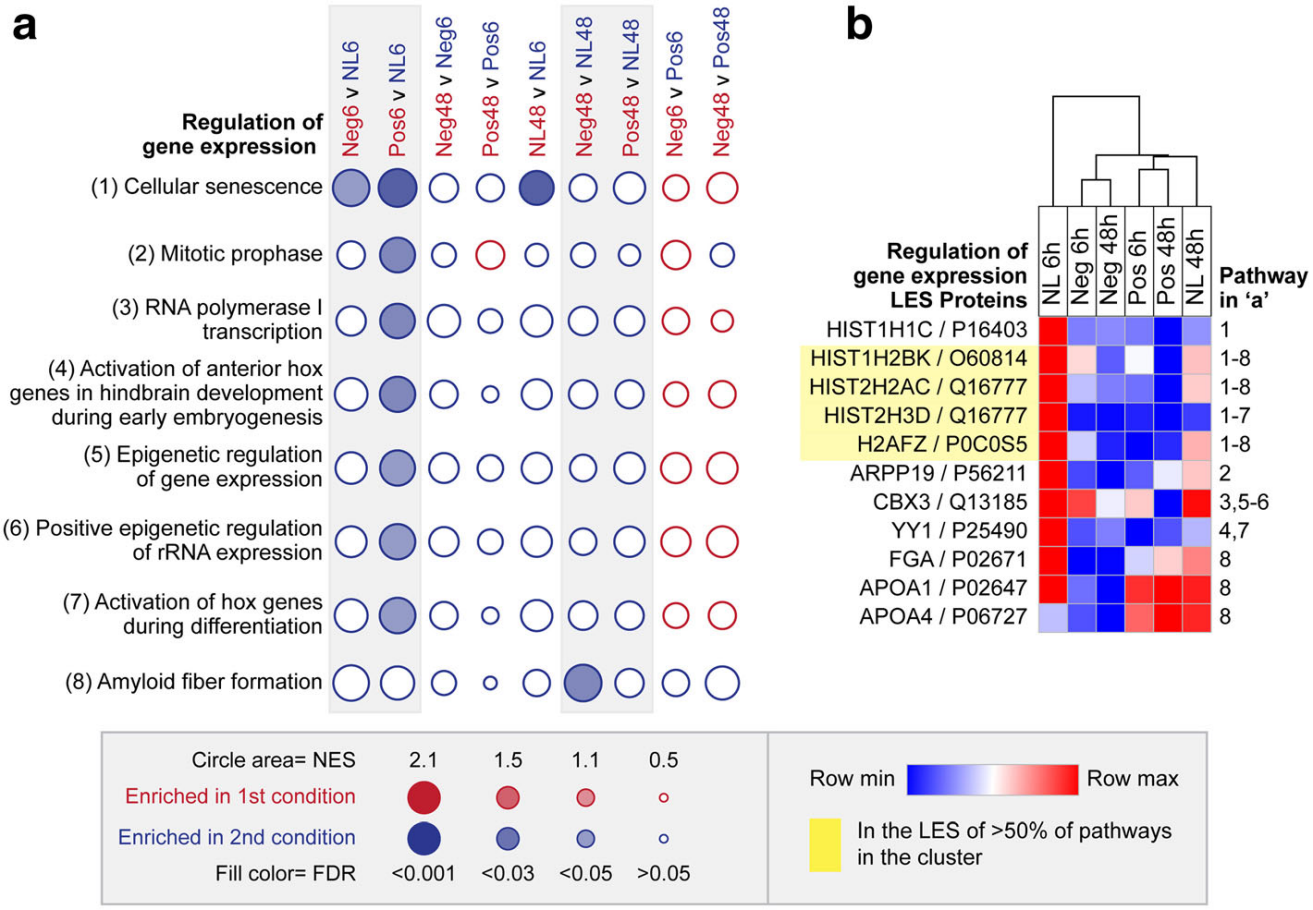
Pathway enrichment in the regulation of gene expression cluster. **a** Bubble plot illustrating the NES and FDR across all pairwise comparisons for the eight enriched pathways in the ‘regulation of gene expression’ cluster. Statistically significant enrichments (FDR < 0.05) are shown as filled circles. **b** Heat map showing the mean LFQ intensity across lens conditions for all LES proteins contributing to the enrichment of one or more pathways in the cluster. Note that, because the ‘cellular senescence’ pathway was significantly enriched across multiple pairwise comparisons, the LES varied depending on the comparison being made. Full details of the LES for each comparison are provided in, Additional file 4 Figure S4

Eight of the 83 implicated pathways were assigned to the ‘regulation of gene expression’ cluster. Pathways in this cluster were down-regulated between the 6 h and 48 h time-points in normally developing chicks, with statistically significant down-regulation occurring for the cellular senescence pathway (consistent with the expected slowing of growth and developmental processes in the no lens group [78]; Fig. 2a). The LES contributing this significant down-regulation (shown in Fig. 2b) was primarily composed of histone proteins from the H2A, H2B, and H3 families (HIST1H2BK, HIST2H2AC, HIST2H3D, and H2AFZ) that form the nucleosome and the H1 family (HIST1H1C) that promotes higher order chromatin structures. Positive and negative lens-wear induced an earlier and stronger down-regulation of these histone proteins, such that pathways from the cluster were significantly down-regulated in the negative and/or positive lens groups relative to the no lens group at 6 h (Fig. 2a). One pathway from the cluster, amyloid fiber formation, was down regulated in the negative lens condition relative to no lens condition after 48 h of lens-wear on P7 (Fig. 2a). This latter pathway’s LES was composed of plasma-related proteins FGA, APOA1, and APOA4 as well as histone proteins (HIST1H2BK, HIST2H2AC, and H2AFZ; Fig. 2b).

### Translation cluster

**Fig. 3:**
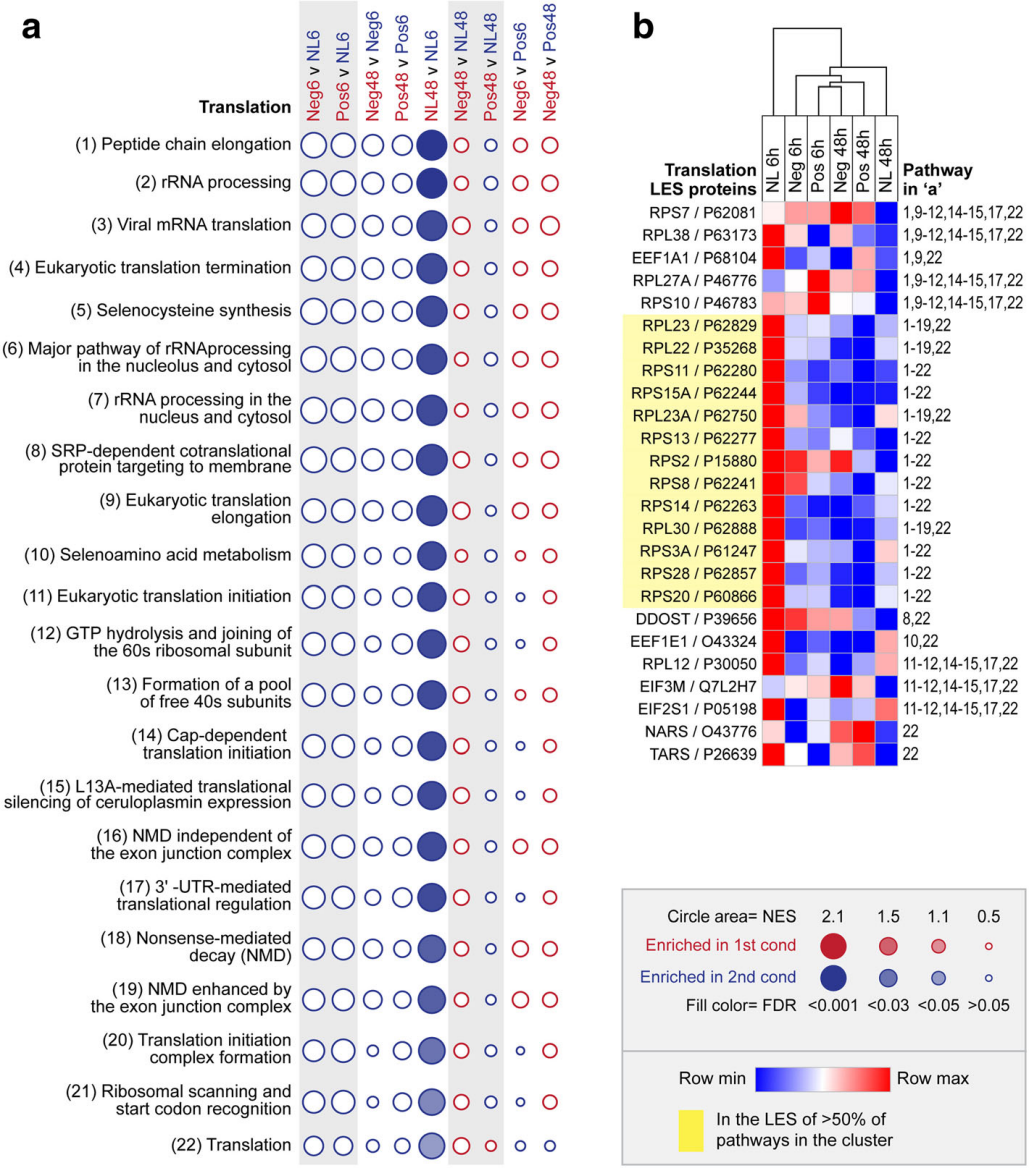
Pathway enrichment in the translation cluster. **a** Bubble plot illustrating the NES and FDR across all pairwise comparisons for the twenty-two enriched pathways in the ‘translation’ cluster. Statistically significant enrichments (FDR < 0.05) are shown as filled circles. **b** Heat map showing the mean LFQ intensity across lens conditions for all LES proteins contributing to the enrichment of one or more pathways in the cluster

Twenty-two of the 83 implicated pathways were assigned to the ‘translation’ cluster (Fig. 3a). The LES of these pathways was comprised of proteins involved in ribosomal translation, including 40s and 60s ribosomal subunits (RPS7, RPS10, RPS11, RPS15A, RPS13, RPS2, RPS8, RPS14, RPS3A, RPS28, RPS20, RPL38, RPL27A, RPL23, RPL22, RPL23A, RPL30, RPL12) and eukaryotic translation initiation and elongation factors (EEF1A1, EEF1E1, EIF3M, EIF2S1; Fig. 3b). These translation-related proteins were highly expressed in the retina/RPE of no lens chicks at the 6 h time-point, and were then significantly down-regulated over time between 6 and 48 h (Fig. 3a-b). Similar to the ‘regulation of gene expression cluster’, positive and negative lens-wear appeared to bring forward the timing of pathway down-regulation; all pathways in the cluster were non-significantly down regulated in the positive and negative lens conditions relative to the no lens condition at the 6 h time-point, and non-significantly down regulated over time between 6 and 48 h in both lens conditions. Consequently, both lens conditions displayed similar expression levels as the no lens animals at the 48 h time-point (Fig. 3a-b).

### Mitochondrial import, Rab GTPase, and integration of energy metabolism clusters

**Fig. 4:**
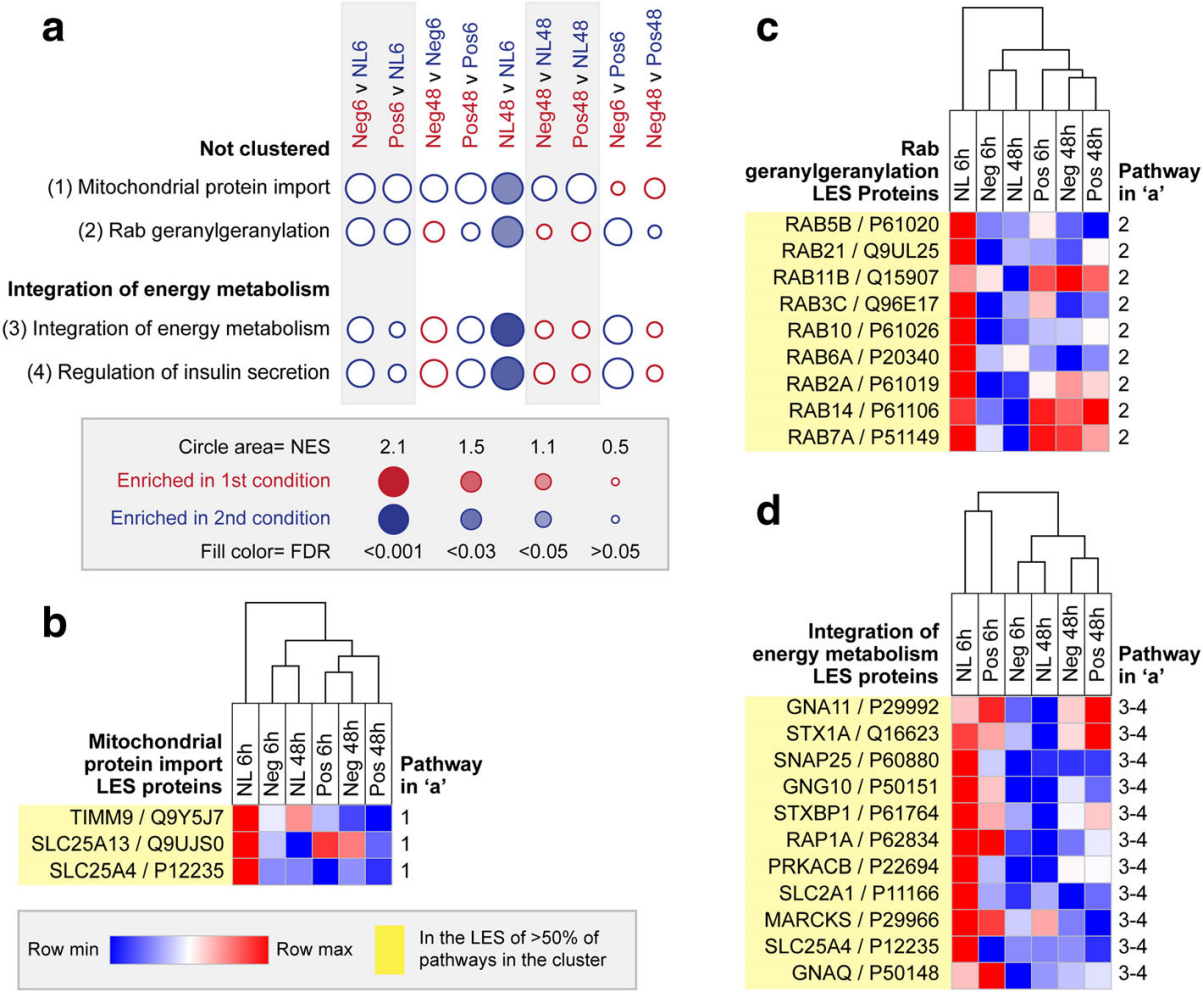
Pathway enrichment in mitochondrial protein import, rab GTPase, and integration of energy metabolism clusters. **a** Bubble plot illustrating the NES and FDR across all pairwise comparisons for the four enriched pathways in the ‘mitochondrial protein import’, rab GTPase and ‘integration of energy metabolism’ clusters. Statistically significant enrichments (FDR < 0.05) are shown as filled circles. Heat maps show the mean LQF intensity across lens conditions for all LES proteins contributing to the enrichment of one or more pathways in the **b** mitochondrial protein import, **c** rab GTPase and **d** integration of energy metabolism clusters

Proteins from mitochondrial protein import, rab geraylgeranylation, integration of energy metabolism, and regulation of insulin secretion pathways were also highly expressed in the retina/RPE of no lens chicks at 6 h, and then down-regulated over time (Fig. 4a-d). The LES of the mitochondrial protein import pathway was comprised of SLC25A13, which is involved in the calcium-dependent exchange of cytoplasmic glutamate with mitochondrial aspartate, SLC25A4, which is involved in translocation of ADP from the cytoplasm into the mitochondria and ATP from the mitochondria into the cytoplasm, and TIMM9 which participates in the import and insertion of proteins into the mitochondrial inner membrane (Fig. 4b). The LES of the rab geranylgeranylation pathway was comprised of rab GTPases involved in intracellular membrane traffic, including several linked to insulin and glucose signalling (Rab5b, Rab7a, Rab10, Rab11b, Rab14 [79–81]) (Fig. 4c). The other two down-regulated pathways, ‘integration of energy metabolism’ and ‘regulation of insulin secretion’ had identical LES, primarily comprised of proteins linked to vesicle exocytosis (STX1A, SNAP25, STXBP1, MARCKS) and G-protein signalling (GNA11, GNG10, GNAQ) (Fig. 4d). Similar to the ‘regulation of gene expression’ and ‘translation’ clusters, positive and negative lens-wear appeared to induce an earlier and stronger down-regulation of proteins from the mitochondrial protein import pathway (Fig. 4a).

### Ion and vascular homeostasis, signal transduction, and solute transport clusters

**Fig. 5:**
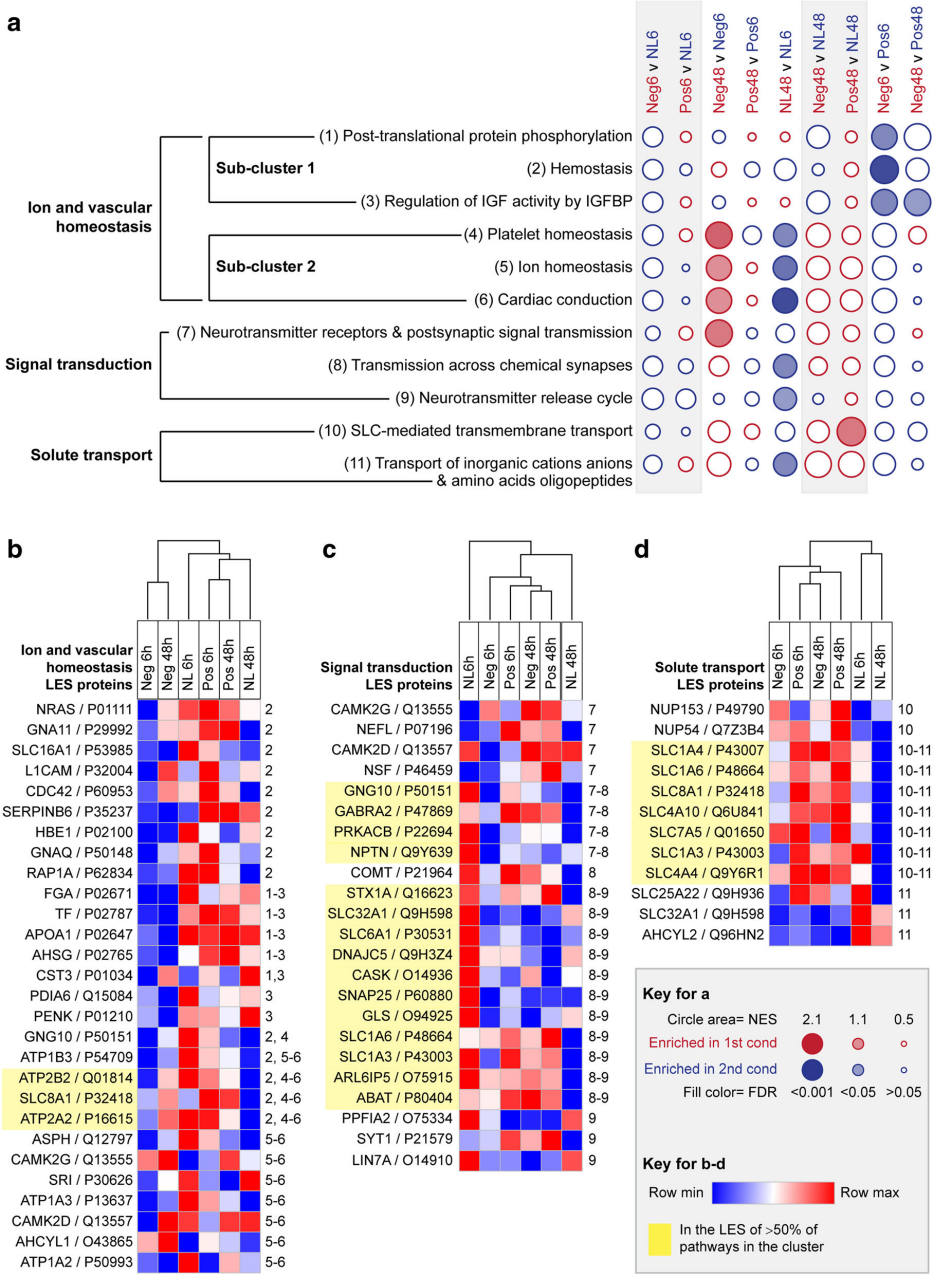
Pathway enrichment in ion and vascular homeostasis, signal transduction, and solute transport clusters. **a** Bubble plot illustrating the NES and FDR across all pairwise comparisons for the eight enriched pathways in the ‘ion and vascular homeostasis’, ‘signal transduction’, and ‘solute transport’ clusters. Statistically significant enrichments (FDR < 0.05) are shown as filled circles. Heat maps show the mean LFQ intensity across lens groups for all proteins that were in the LES of one or more enriched pathways in the **b** ion and vascular homeostasis, **c** signal transduction and **d** solute transport clusters. Note that the LES varied across pathways that were significantly enriched in multiple lens groups (i.e., several of the ion and vascular homeostasis pathways). Full details of the LES for each comparison are provided in, Additional file 4 Figure S5

The final three clusters of pathways, ‘ion and vascular homeostasis, ‘signal transduction’, and ‘solute transport’, displayed distinct expression patterns across the three lens conditions. The three pathways related to vascular homeostasis were down-regulated in the negative relative to positive lens group at 6 h (sub-cluster 1 in Fig. 5a). One of these pathways (regulation of IGF by IGFBP) was also down regulated in the negative relative to positive lens group at the 48 h. Four of the LES proteins common to these three pathways were plasma-associated (FGA, TF, APOA1, and AHSG; Fig. 5b). FGA is involved in blood clotting, TF transports iron to proliferating cells, APOA1 is a major component of high density lipoprotein (HDL), and ASHG is involved in tissue development. The most significantly down regulated pathway in this sub-cluster, ‘hemostasis’, also had the largest number of LES proteins. Here, a range of other proteins including GTPases (CDC42, RAP1A, NRAS), G-proteins (GNA11, GNAQ, GNG10), and ion transport proteins (particularly those involved in calcium exchange; ATP2B2, ATP2A2, SLC8A1) were implicated in the LES. Several of these ion transport proteins were also implicated in the ion homeostasis pathways that were down-regulated between 6 and 48 h in normally developing chicks, and up-regulated between 6 and 48 h in chicks wearing negative lenses (Sub-cluster 2 in Fig. 5a). In addition to proteins involved in calcium exchange, the LES of these ion homeostasis pathways included three Na^+^/K^+^-ATPase subunits (ATP1A2, ATP1A3, and ATP1B3) that are expected to be involved in the maintenance of cell membrane potentials [82].

Two signal transduction pathways were down-regulated between 6 and 48 h in normally developing no lens chicks, and a related pathway was up-regulated over the same time period in chicks wearing negative lenses (Fig. 5a). The LES of the two pathways that were down-regulated during normal development included a range of proteins involved in glutamate and GABA signalling (Fig. 5c). The glutamate-related proteins included glutaminase (GLS), which catalyses the conversion of glutamine to glutamate, and the sodium and potassium dependent glutamate transporters EAAT1 (SLC1A3) and EAAT4 (SLC1A6). The GABA-related proteins included GABA(A) Receptor Subunit Alpha-2 (GABRA2), transporters involved in GABA removal from the synaptic cleft and uptake into synaptic vesicles (GAT1, encoded by SLC6A1, and VGAT, encoded by SLC32A1), and the GABA Aminotransferase (ABAT), which catalyses the conversion of GABA to succinate semialdehyde. The LES of the related signal transduction pathway that was up-regulated between 6 and 48 h in chicks wearing negative lenses contained only one GABA receptor sub-unit (GABRA2), with the remainder of the LES comprised of Neurofilament light polypeptide (NEFL) that forms a major component of axon cytoskeletons, N-ethylmaleimide-sensitive factor (NSF) that is involved in membrane fusion, a catalytic subunit of cAMP-dependent protein kinase (PRKACB), and Neuroplastin (NPTN), which is involved in cell-cell interactions (Fig. 5c).

The final cluster was related to solute transport (Fig. 5a). Here, the ‘transport of inorganic cation, anions, and amino acids/oligopeptides’ pathway was down-regulated between 6 and 48 h in no lens chicks. The LES of this pathway included several of the glutamate, GABA, and Na^+^/Ca^++^ transporters from the ion and vascular homeostasis and signal transduction clusters, as well as bicarbonate transporters (SLC4A10, SLC4A4), an amino acid exchanger (SLC7A5), and a mitochondrial glutamate carrier (SLC25A22) (Fig. 5d). The second pathway from the solute transport cluster, ‘SLC-mediated transmembrane transport’, was up-regulated in the positive lens group relative to the no lens group at 48 h. This pathway had a highly similar LES to the solute transport pathway that was down-regulated over time in the no lens group, including glutamate transporters (SLC1A6, SLC1A3), bicarbonate transporters (SLC4A10, SLC4A4), and sodium-calcium (SLC8A1) and amino acid (SLC1A4, SLC7A5) exchangers (Fig. 5d).

### Cross-pathway interactions

**Fig. 6:**
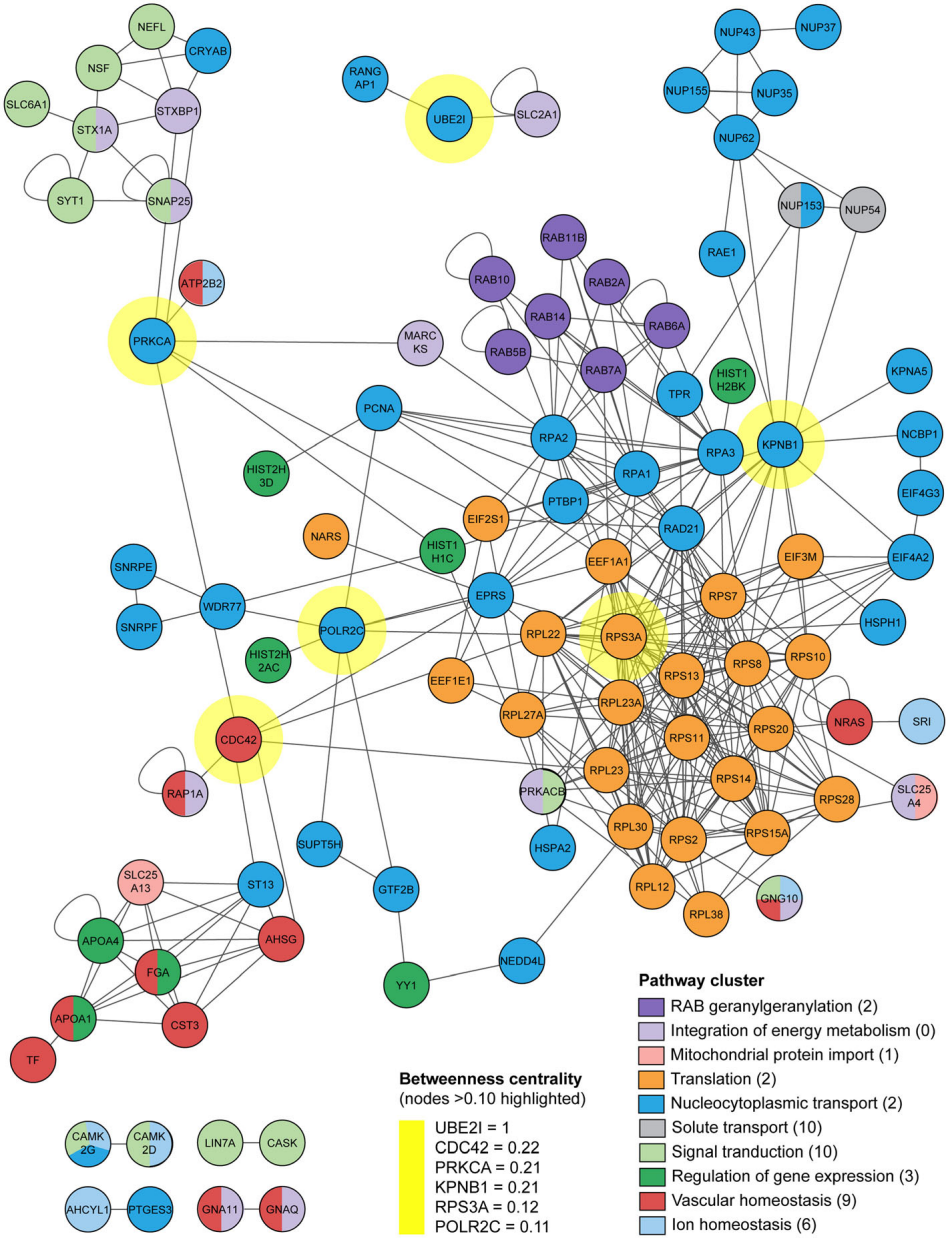
Interactions between leading edge subset proteins. A protein-protein interaction (PPI) network for the LES proteins from all significant pathway enrichments was generated in Cytoscape. Proteins in the network are coloured according to pathway cluster from the GSEA, and connections between proteins indicate a protein-protein interaction. Topological analysis was used to identify proteins with a high degree of betweenness centrality (i.e., proteins that connect subnetworks within the interaction diagram). These key proteins are highlighted in yellow. LES proteins with no interactions are not shown in the diagram. The number of LES proteins not shown for each cluster is indicated in parenthesis after the cluster name in the ‘pathway cluster’ key

To better understand interactions between the nine implicated pathway clusters, we created a protein-protein interaction (PPI) network for the LES proteins from all significant pathway enrichments. This PPI network demonstrated extensive interactions between the LES from different pathway clusters (Fig. 6). Topological analysis highlighted several nucleocytoplasmic transport proteins (PRKCAB, POLR2C, UBE2I and KPNB1), the hemostasis pathway protein CDC42, and the ribosomal protein RPS3A as having potential to exert a high degree of control over the interactions of other nodes in the network (termed ‘betweenness centrality’) [83].

## Discussion

This study has compared protein abundance in the chick retina/RPE during normal development and lens-induced myopia and hyperopia. We specifically aimed to elucidate how lens-wear affects normal developmental processes and to identify pathways showing bidirectional and/or distinct expression responses across the lens conditions as such pathways are likely to be involved in directional ocular growth, and may be of interest as therapeutic targets. Seventy-one Reactome pathways linked to cell development and neuronal maturation were differentially enriched between 6 h (P5) and 48 h (P7) in no lens animals. Lens-wear disrupted most of these normal developmental changes, and induced lens-specific shifts in a subset of pathways related to neurotransmission (particularly GABA and glutamate signalling), calcium homeostasis, and ATP-driven ion transport.

### Developmental pathways in no lens chicks

The range of protein abundance changes that we identified between 6 and 48 h in no lens chicks was broadly consistent with maturation and the consequent slowing of cell growth as the retina develops post-hatch [78, 84]. Histone and translational pathways were down-regulated between 6 and 48 h, and nucleoporin and heat shock-related pathways were up-regulated over the same time period. These pathways are closely linked to developmental processes, such as histone protein synthesis that is needed to package replicated DNA into chromatin during cell cycle progression [85, 86] and the ribosome content of a cell which determines its capacity for protein production and associated growth [87–89]. Heat shock [90] and nucleocytoplasmic transport pathways [91, 92] are also involved in the dynamic regulation of gene and protein expression during development, and both of these pathways are closely tied to ribosomal biogenesis and translational processes [93–95].

Proteins from a range of ion homeostasis and signalling pathways were also down-regulated between P5 and P7 in the no lens group, as would be expected in chicks with plano refraction undergoing normal post-hatch refinement of neuronal networks [78]. The LES of these pathways was composed primarily of GABA and glutamate transporters and receptor subunits, rab GTPases linked to insulin and glucose signalling, and Ca^++^ and Na^+^/K^+^ transporting ATPases. Proteins from calcium-related signalling pathways were also implicated, including those involved in vesicle exocytosis and G-protein signalling. Past research has demonstrated close interconnections between these GABA, glutamate, glucose, insulin, and calcium signalling pathways during neuronal development [96–99].

### Positive and negative lenses disrupt the timing of normal developmental shifts

Up-regulation of nucleocytoplasmic transport pathways between 6 and 48 h was preserved in the positive lens group, however, lens-wear (of both signs) disrupted the timing, strength, and/or direction of all of the other expression shifts seen in normally developing no lens animals. In most cases (i.e., for pathways from ‘regulation of gene expression’, ‘translation’, and ‘mitochondrial protein import’ clusters) this appeared to occur because lens-wear of both signs induced a premature down-regulation of pathway expression relative to that seen in no lens animals (i.e., the pathways were down-regulated in lens relative to no lens groups at 6 h, and then further down-regulated over time between 6 and 48 h in both lens groups). These non-specific responses primarily affected pathways linked to basic cellular processes (such as histone proteins, ribosomal subunits, and translation elongation factors) which are known to be down-regulated in response to multiple types of cellular stress [e.g. 85, 100, 101, 102]. Such stress-related expression responses are usually associated with a slowing of the cellular growth rate [100, 101], which helps to explain the pattern of overlap with pathway down-regulation in no lens chicks where normal post-hatch developmental processes are also expected to be slowing down [78, 84]. The general stress response is aimed at increasing cellular tolerance limits under adverse conditions, and allowing time for adaptive responses specific to the stressor to re-establish homeostasis [102]. Thus, although these non-specific shifts presumably reflect some secondary effect of lens-wear (such as physiological stress resulting from blur or peripheral occlusion by the Velcro ring), they could still be involved in priming the system for growth-specific changes (for example by down-regulating ribosomal biogenesis in order to conserve metabolic energy for use in homeostatic lens-specific adaptations to neurotransmission).

### Lens-wear induces distinct expression patterns in pathways related to neurotransmission and ion homeostasis

In addition to these non-specific changes, lens-wear induced distinct growth-specific expression patterns in a small number of pathways primarily related to neurotransmission and ion homeostasis. As discussed briefly above, ‘Transmission across chemical synapses’ and ‘neurotransmitter release cycle’ pathways were down-regulated between P5 and P7 in normally developing chicks. Many of the core proteins in these pathways were involved in glutamatergic (GLS, SLC1A3, SLC1A6) or GABAergic (GABRA2, SLC6A1, SLC32A1, ABAT) signalling. Previous studies have similarly demonstrated changes in the expression of GABA and glutamate pathway genes and proteins in the retina during normal post-natal emmetropization, including GABA_A_ receptor subunits [103], the ABAT GABA aminotransferase [57], and glutamate carrier subunits [69].

In contrast to the no lens condition, the ‘neurotransmitter receptors and postsynaptic signal transmission’ pathway was enriched between 6 and 48 h in the myopia induction condition, suggesting an up-regulation of aspects of neurotransmission (particularly GABA signalling) during the mid to late stages of refractive compensation to -10D lenses. The core proteins responsible for this enrichment included the GABA_A_ receptor subunit alpha 2 (GABRA2) and N-ethylmaleimide-sensitive factor (NSF), which is involved in GABA_A_ receptor subunit trafficking to the plasma membrane [104]. Several proteins linked to the regulation of synapse organization and strength were also implicated. Some previous studies have reported down-regulation of GABA-related genes or retinal GABA content during lens and occlusion myopia induction [51, 58, 105, 106], however, most reports are consistent with the increase in GABA pathway signalling suggested here. Specifically, the present findings are in agreement with previous reports of up-regulation of the NSF protein during lens-induced myopia in mouse [63], and up-regulation of the GABA_A_ receptor mRNA and protein [107] and increased retinal GABA content during lens-induced myopia in guinea pigs [107, 108]. This pattern of expression responses is also consistent with drug studies showing that GABA receptor antagonists inhibit myopia development [105, 109, 110]. Similarly, the GABA_C_ receptor antagonist TPMPA enhanced the protective effect of short periods of normal vision on myopia development in chick, and the GABA_A/C_ agonist Muscimol inhibited the protective effect of normal vision [111].

Changes to the expression of GABA, glutamate, and synaptic proteins between 6 and 48 h in no lens and negative lens groups are suggestive of activity-dependent ‘tuning’ of retinal signalling [112]. Calcium influx into cells provides a ubiquitous ‘activity signal’ that is crucial for such synaptic plasticity and, in accordance with this, pathways related to the maintenance of ion homeostasis displayed the same expression patterns as the signal transduction pathways in no lens and negative lens groups. Specifically, ‘ion homeostasis’, ‘platelet homeostasis’ and ‘cardiac conduction’ pathways were up-regulated between 6 and 48 h in the negative lens group, and down-regulated between 6 and 48 h in the no lens group. The core proteins in these pathways were predominantly related to calcium regulation, including Ca^++^ transporting ATPases (ATP2B2 and ATP2A2), the NCX1 Na^+^/Ca^++^ exchanger (SLC8A1), sorcin (SRI) [113], aspartate beta-hydroxylase (ASPH) [114], and both the gamma and delta subunits of calcium/calmodulin-dependent protein kinase II (an enzyme complex known to drive activity-dependent changes in synapse strength [115, 116]). GABA and glutamate transporters are reliant on Na^+^/K^+^-ATPase to generate the ion gradients that drive transmitter uptake [117, 118] and, in accordance with this, Na^+^/K^+^-ATPase subunits were also among the core proteins implicated in the ion homeostasis pathways. Interestingly, Na^+^/K^+^-ATPase inhibition with Ouabain has been shown to prevent compensation to lenses in chick [119]. Our previous RNA-sequencing study has also identified up-regulation of mitochondrial oxidative phosphorylation genes in the retina/RPE/choroid of chicks of the same age following 2 days of negative lens-wear [57]. Considered together with the concurrent up-regulation of ATP-dependent ion transporters and GABA-related signalling proteins during negative lens-wear in the present study, these findings suggest a close relationship between shifts in neurotransmission, cellular ion homeostasis, and the associated utilization of cellular energy resources during myopia induction.

Negative lens-wear also induced a down-regulation of several pathways related to ion and vascular homeostasis at 6 h (‘post-translational protein phosphorylation’, ‘hemostasis’, and ‘regulation of IGF by IGFBP’), resulting in their enrichment in the positive relative to the negative lens group. The core proteins in these pathways were disparate, but notably included Ca^++^ transporting ATPases (ATP2B2 and ATP2A2) and the NCX1 Na^+^/Ca^++^ exchanger (SLC8A1), as well as three of the four plasma-related proteins recently shown to be up-regulated in chick vitreous following negative lens-wear relative to positive lens-wear (APOA1, TF, and CST3) [120]. The plasma-related proteins remained down-regulated in the negative lens group at 48 h, while calcium-related proteins were up-regulated between 6 and 48 h (as discussed above). This shift in calcium regulation between the early and late time-points is notable, and perhaps related to the divergent expression of glutamate and GABA pathway mRNA previously reported between 6 h and 72 h time-points in the chick negative lens model [51].

Positive lens-wear, by comparison, inhibited the normal down-regulation of solute transport proteins between 6 and 48 h. This resulted in enrichment of the ‘SLC-mediated transmembrane transport’ pathway in the positive lens group relative to the no lens group at 48 h. The core proteins driving this enrichment included EAAT1 (SLC1A3) and EAAT4 (SLC1A6) glutamate transporters. EAAT1 (also known as GLAST) is the predominant glutamate transporter in the retina. It is localized on Muller cells [121], and is essential for the glutamate-glutamine cycle [122]. EAAT4 is localized on the photoreceptor outer segments [123]. Both of these transporters clear glutamate from the extracellular space, a function important for maintaining an accurate representation of the light signal. Also implicated were the sodium-driven chloride bicarbonate exchanger NCBE (SLC4A10) and the sodium-bicarbonate cotransporter NBCE1 (SLC4A4). NCBE is thought to act together with KCC2 to maintain low intracellular chloride in OFF bipolar cell dendrites and ON bipolar cell terminals, thus supporting the hyperpolarizing action of GABA [124, 125]. NCBE knockout mice display decreased contrast sensitivity and visual acuity, and deceased ON bipolar cell activity [124]. NCBE1 is expressed on Muller cells and apical membrane of the RPE, and is thought to regulate light-induced extracellular alkalosis [126].

These expression changes in the positive lens group are suggestive of differences in the sensitivity of retinal signalling pathways, particularly at photoreceptor-bipolar synapses. It is possible that these expression changes are an adaptation to the decrease in expression of photoreceptor proteins that we have previously reported following 6 h of positive lens-wear in this dataset [66]. The need for signalling adaptations at the photoreceptor-bipolar level is predicted by our RIDE model [77] and consistent with pharmacological and gene knockout studies showing that changes to the balance of ON and OFF bipolar signalling can affect post-natal ocular growth and the induction of optically-induced refractive errors [38, 127–133]. Recent genetic studies have also strongly implicated photoreceptor and bipolar cell signalling in the development of refractive errors in humans [75, 76].

## Conclusions

This study has shown that positive and negative lens-wear induces the premature down-regulation of a range of basic housekeeping pathways in the retina/RPE. These non-specific expression shifts are presumably driven by the secondary effects of lens-wear, and could represent a general stress response with the potential to promote lens-specific homeostatic adaptations. A smaller number of pathways displayed distinct expression profiles across the lens conditions. These latter findings provide support for past research suggesting that GABAergic signalling is up-regulated during myopia induction [63, 105, 107–111]. Changes in the expression of ion transport and binding proteins, particularly those related to calcium regulation and Na-K-ATPase, further supports the existence of theoretically expected links between shifts in neurotransmission, ion homeostasis, and energy requirements [57, 58, 106] during lens-induced myopia. Previous x-ray microanalysis and microarray studies have reported retinal hyperosmolarity and similar pathway expression shifts following occlusion myopia in chick [58, 134, 135], suggesting a similar molecular basis for lens and occlusion myopia models (in line with past research e.g. [31, 70]). Our findings also provide novel evidence for changes to glutamate and bicarbonate/chloride transport during hyperopia induction, with potential implications for signalling at the photoreceptor-bipolar synapse. Overall, these findings reflect a key role for perturbed neurotransmission and associated ion homeostasis in optically induced ocular growth changes, as predicted by our RIDE model [77].

## Methods

### Generation and pre-processing of LC-ESI-MS/MS data

The methods used to generate the LC-ESI-MS/MS data analysed in this study have been described in our previous publication [66]. Briefly, 31 male chicks (Leghorn/New Hampshire) were assigned to a lens condition (right eye + 10 dioptre (D), right eye -10D, or no lens) on P5. Lenses were attached with Velcro, and lensing was staggered so that subsequent data collection time-points were circadian matched. Separate no lens controls were used because monocular treatments can affect blood flow [136, 137], refraction and axial length [28, 138], and gene and protein expression in the fellow eye [138, 139]. Furthermore, we used right eyes only because chicks are oriented in the embryo such that the left eye is occluded while the right eye receives light stimulation. This produces developmental asymmetries within the left and right visual pathways of young male chicks following hatching [140–142].

Following 6 h (P5) or 48 h (P7) of lens-wear, chicks were anaesthetized (ketamine, 45 mg/kg; xylazine, 4.5 mg/kg i.m.) and their right eye refraction and axial dimensions were measured via manual retinoscopy (Keeler, Vista Diagnostic Instruments, Windsor, UK) and a-scan ultrasonography (7 MHz probe; A-Scan III, TSL: Teknar, Inc., St Louis, MO). Biometric data was not recorded for the unused left eyes in order to minimize time under anaesthesia prior to tissue collection. The chicks were then euthanized via decapitation, and the retina/RPE was immediately collected from the right eye for LC-ESI-MS/MS analysis. All procedures adhered to the ARVO Statement for the use of Animals in Ophthalmic and Vision Research and were conducted in accordance with approved La Trobe University Animal Ethics Committee protocols (AEC14–60).

Retina/RPE samples from 5 chicks per condition were analysed individually using LC-ESI-MS/MS, with the exception of the no lens 48 h condition where tissue from 6 chicks was profiled. Samples were re-suspended in digestion buffer (8 M urea, 50 mM ammonium bicarbonate, 10 mM DTT) and incubated for 5 h. Samples were centrifuged, and the soluble fraction was used for protein concentration determination. Each sample (50 μg protein) was adjusted to 100 μl with digestion buffer, and 55 mM iodoacetamide was then added to alkylate thiol groups for 35 min. The preparation was diluted to 1 M urea with 25 mM ammonium bicarbonate, and trypsin was added to a 5 μM final concentration for overnight digests. The digests were acidified with 1% (*v*/v) trifluoroacetic acid (TFA) and the peptides desalted on SDB-XC Empore StageTips (3 M Company, St. Paul, MN) and dried in a SpeedVac centrifuge as described previously [143]. Peptides were reconstituted in 0.1% TFA and 2% acetonitrile (ACN), loaded onto trapping columns (C_18_ PepMap 100 μm ID × 2 cm, Thermo-Fisher Scientific, San Jose, CA) at 5 μl/min for 6 min, and washed for 6 min before switching the pre-column in line with the analytical column (Vydac MS C_18_, 3 μm, 300 Å and 75 μm ID × 25 cm, Grace Pty. Ltd., Columbia, MD). The separation of peptides was performed at 300 nl/min using a linear ACN gradient of buffer A (0.1% formic acid, 2% ACN) and buffer B (0.1% formic acid, 80% ACN), starting at 5% buffer B to 55% over 120 min. Data were collected on an Orbitrap Elite (Thermo-Fisher Scientific) in Data Dependent Acquisition mode using m/z 300–1500 as MS scan range. Collision-induced dissociation (CID) MS/MS spectra were collected for the 20 most intense ions per MS scan. The dynamic exclusion parameters used were: repeat count 1, duration 90 s and the exclusion list size was set at 500 with early expiration disabled. Other instrument parameters for the Orbitrap Elite were as follows: MS scan at 120000 resolution, maximum injection time 150 ms, automatic gain control (AGC) target 1 × 10^6^, CID at 35% energy for a maximum injection time of 150 ms with AGT target of 5000. The instrument was operated in dual analyser mode with the Orbitrap analyser being used for MS and the linear trap being used for MS/MS.

Identification and label-free quantification of proteins was performed on the raw output files from LC–ESI-MS/MS using MaxQuant (Version 1.5.1.6; [144, 145]) and the Andromeda search engine (September 2016 *Gallus gallus* Uniprot FASTA database). Peptides with a minimum of seven amino-acid length were considered, and the required FDR was set to 1% at the peptide and protein level. Protein group intensity values were normalized using the MaxLFQ algorithm [145] and log base 2 transformed. Flagged protein groups and protein groups with > 40% missing values in any condition were filtered from the results. The remaining missing values were imputed using a QRLIC/SVD approach (imputeLCMD R package v2.0; [146]), and the data were then normalized using the LIMMA Cyclic Loess function (package v3.30.12; [147]). Finally, to enable the use of curated human Reactome Gene Matrix Transposed (GMT) files for pathway analyses, high confidence human orthologs for each Uniprot Accession in the dataset were identified using InParanoid (v8.0) [148].

### Gene set enrichment analysis

*Homo sapiens* GMT files for Reactome pathways (Release 37 [149]) were obtained from the Bader lab on 17-06-2017 [150]. These GMT files and the dataset of normalized log transformed LFQ intensity values mapped to human ortholog UniProt IDs were imported into the javaGSEA desktop app [151]. GSEA was used to assess Reactome pathway expression in lens relative to no lens groups at each time-point, negative relative to positive lens-groups at each time-point, and within each lens-group over time.

The first step in GSEA involves ranking all proteins in the dataset. In the present study, proteins were ranked by the difference between group means scaled by the standard deviation (the default GSEA ‘signal to noise’ metric). Once the dataset was ranked, the enrichment score (ES) for each pathway was calculated by walking down the ranked list of proteins, increasing the cumulative enrichment score when a protein was in the pathway, and decreasing it when it was not. The final ES for each pathway is the maximum deviation from zero of the cumulative score when walking down the ranked list and it can be interpreted as a weighted Kolmogorov–Smirnov statistic [74, 151]. The significance of pathway ES’s was calculated by gene set permutation. Table 2 provides a summary of the GSEA output metrics and their use in the present study.

**Table 2:** Description of GSEA metrics

Metric	Description
Enrichment score (ES)	Reflects the degree to which proteins in a pathway are over-represented at the top or bottom of the ranked protein list.
Normalized enrichment score (NES)	A normalized version of the enrichment score that accounts for differences in pathway size. This is the primary statistic for comparing results across pathways.
False discovery rate (FDR)	The probability that a pathway with a given NES represents a false positive finding. An FDR cut-off of 5% was used to determine statistical significance in the present study.
Leading edge subset (LES) proteins	Proteins that appear in the ranked list at or before the point that the running ES reaches its maximum deviation from zero. These are the core proteins driving pathway enrichment. In the present study, LES proteins were used to group similar pathway findings into clusters (thus reducing redundancy and aiding with interpretation).

Pathways with similar leading edge subject (LES) proteins driving their enrichment were clustered together using the Cytoscape Enrichment Map app [150] to facilitate interpretation and reduce redundancy (Additional file 3: Figure S1-S2). GSEA results (shown as bubble plots [152]) and LES analysis results (shown as heat maps) are presented separately for each pathway cluster in the results section. Hierarchical clustering (Euclidean Average Linkage method [153]) of the lens groups in each heat map was performed using the Broad Institute Morpheus web app [154].

### Protein-protein interaction networks

A protein-protein interaction (PPI) network for the LES proteins from all significant pathway enrichments was generated using the Protein Interaction Network Analysis platform (PINA) Cytoscape plugin [155]. This platform integrates data from six different protein-protein interaction databases (IntAct, MINT, BioGRID, DIP, HPRD and MIPS MPact). The Cytoscape Network Analyser plugin [156] was then used to identify nodes with a high degree of betweenness centrality (i.e., proteins that connected dense subnetworks within the interaction diagram).

## Additional files

Additional file 1: **Table S1.** Biometric measures (refraction, axial length, and vitreous chamber depth) for each sample. (XLSX 10 kb)

Additional file 2: **Table S2.** Pre-processed LC-ESI-MS/MS dataset. (XLSX 608 kb)

Additional file 3: **Figure S1-S2.** GSEA pathway clusters created using the Cytoscape Enrichment Map app. (PDF 643 kb)

Additional file 4: **Figure S3-S5.** Leading edge subset proteins for pairwise enrichments in the nucleocytoplasmic transport, regulation of gene expression, ion and vascular homeostasis, signal transmission, and solute transport clusters. (PDF 980 kb)
