# Supplementary material for: Short term optical defocus perturbs normal developmental shifts in retina/RPE protein abundance
Source: BMC Dev Biol. 2018 Aug 29;18:18. doi: 10.1186/s12861-018-0177-1 (PMC6116556; doi:10.1186/s12861-018-0177-1)
Supplement: Supplementary file 3 — Figure S1-S2. GSEA pathway clusters created using the Cytoscape Enrichment Map app. (PDF 643 kb) [file 12861_2018_177_MOESM3_ESM.pdf]

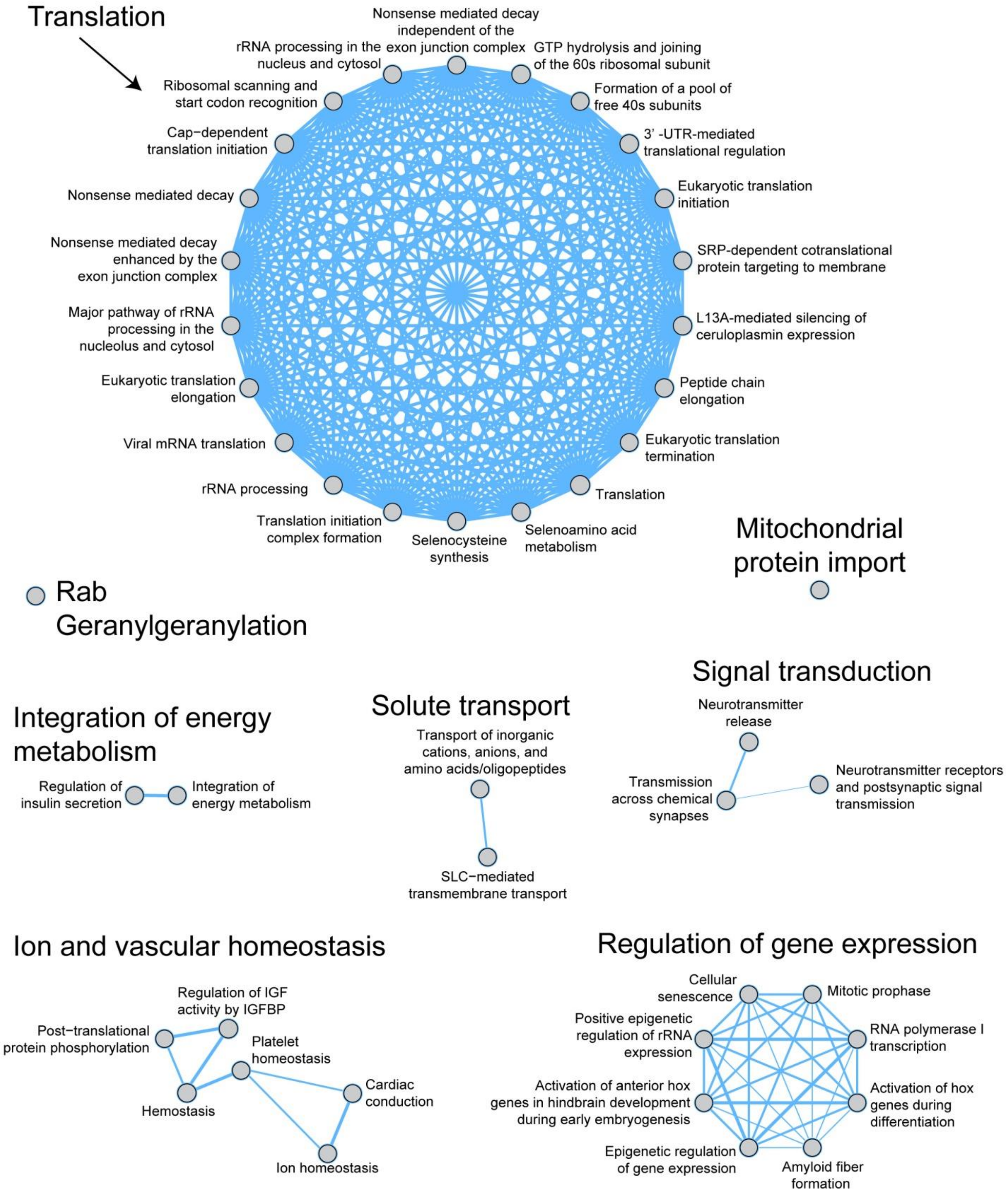

Figure S1. GSEA pathway clusters (part 1). The leading edge subset (LES) proteins driving all significant pathway enrichments were collated into a single file, and this file was then used to identify pathway clusters with an overlap co-efficient of >0.5 in the Cytoscape Enrichment Map app (http://www.baderlab.org/Software/EnrichmentMap). The results of the cluster analysis are shown in this figure, with nodes representing pathways and connecting lines representing common proteins within the LES of two pathways (thicker lines = more overlap). GSEA results are presented separately for each pathway cluster in the results section. Due to space constraints, the remaining pathway cluster is shown on the following page in Supplementary Figure S2.

Nucelocytoplasmic transport

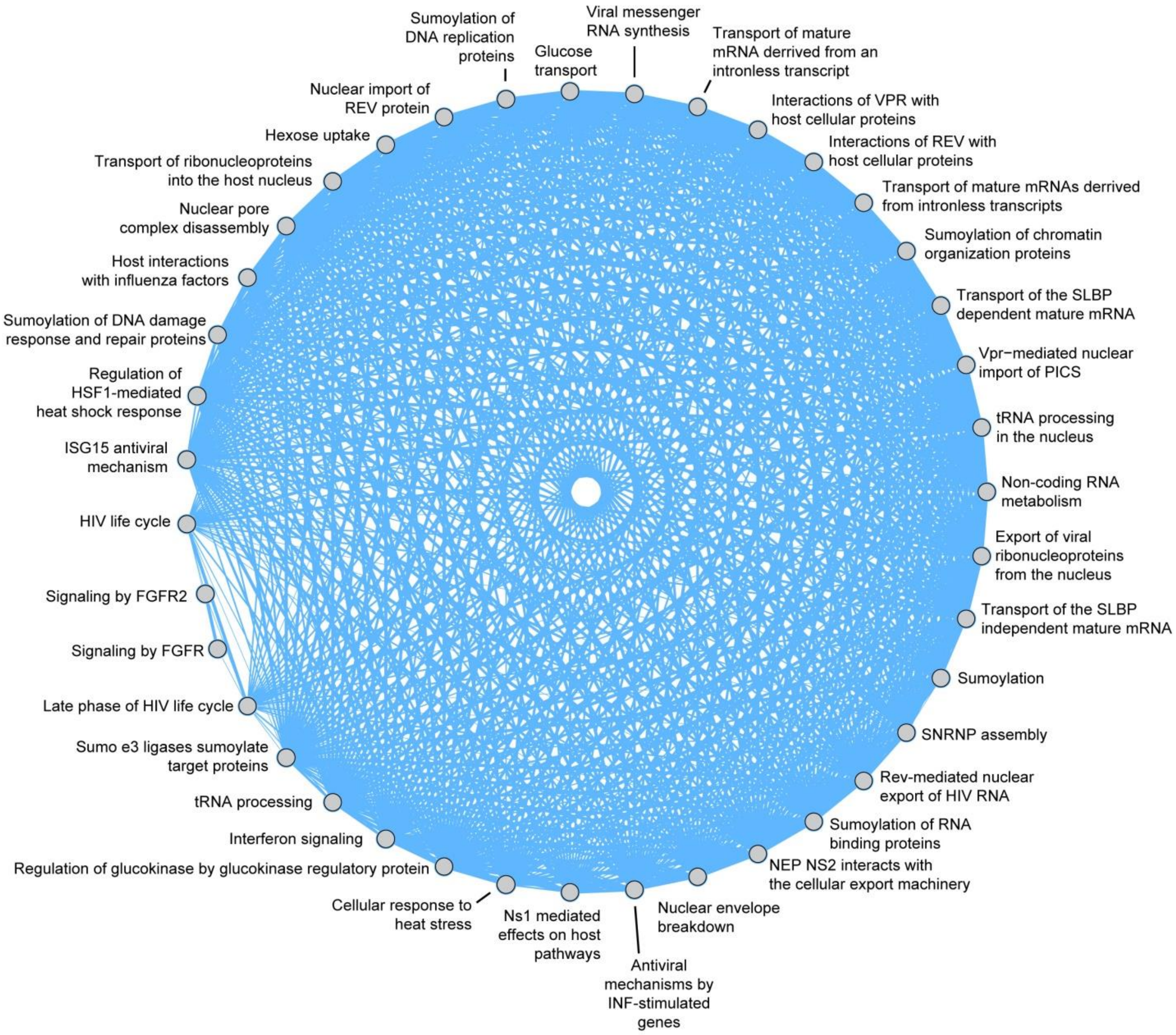

Figure S2. GSEA pathway clusters (part 2). The leading edge subset (LES) proteins driving all significant pathway enrichments were collated into a GMT file, and this file was then used to identify pathway clusters with an overlap co-efficient of >0.5 in the Cytoscape Enrichment Map app. The results of the cluster analysis are shown in this figure, with nodes representing pathways and connecting lines representing common proteins within the LES of two pathways (thicker lines = more overlap). GSEA results are presented separately for each pathway cluster in the results section of the manuscript.
