# Supplementary material for: Short term optical defocus perturbs normal developmental shifts in retina/RPE protein abundance
Source: BMC Dev Biol. 2018 Aug 29;18:18. doi: 10.1186/s12861-018-0177-1 (PMC6116556; doi:10.1186/s12861-018-0177-1)
Supplement: Supplementary file 4 — Figure S3-S5. Leading edge subset proteins for pairwise enrichments in the nucleocytoplasmic transport, regulation of gene expression, ion and vascular homeostasis, signal transmission, and solute transport clusters. (PDF 980 kb) [file 12861_2018_177_MOESM4_ESM.pdf]

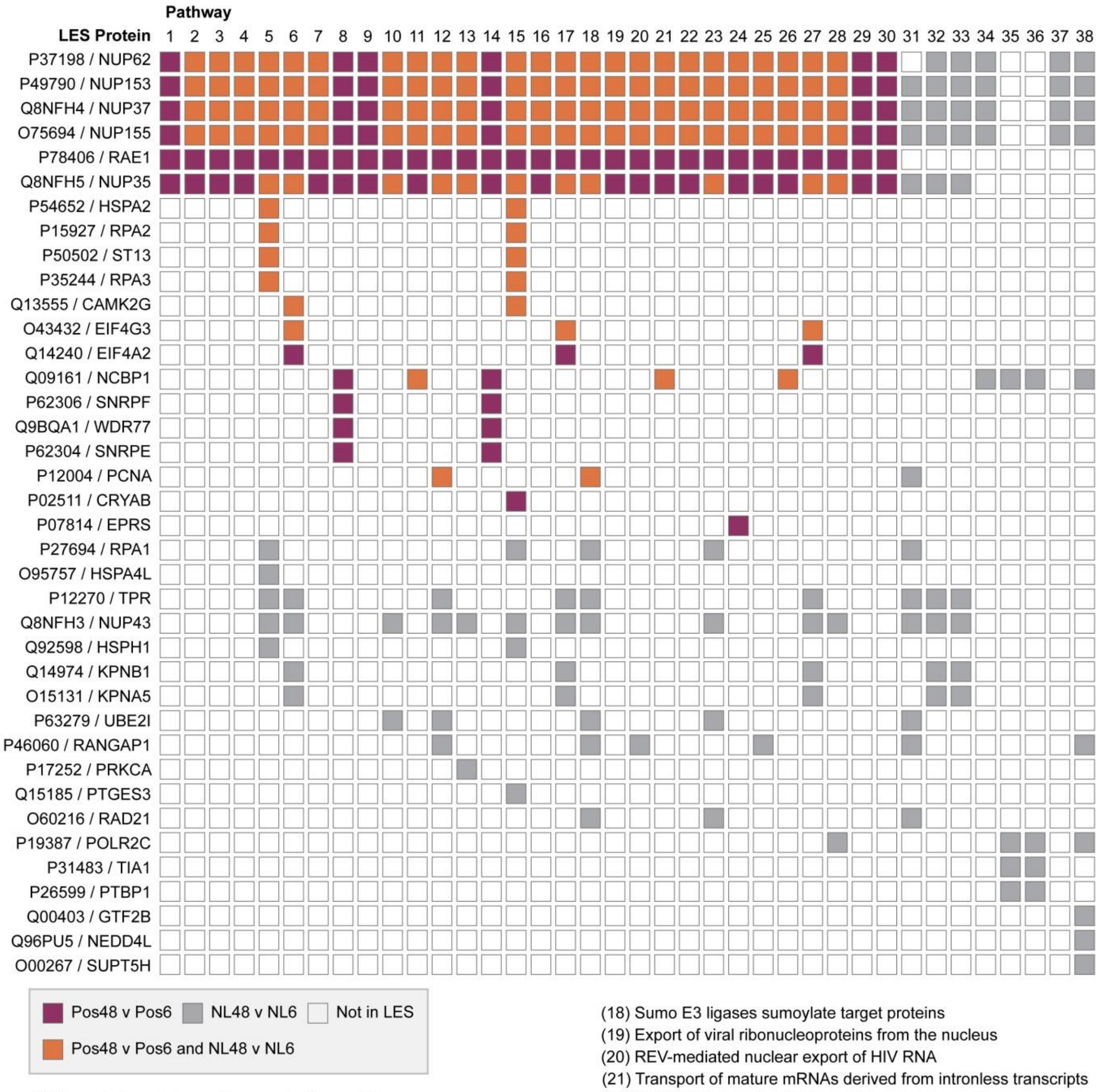

(1) Sumoylation of chromatin organization proteins
(2) Regulation of glucokinase by glucokinase regulatory protein
(3) VPR-mediated nuclear import of pics
(4) Nuclear import of REV protein
(5) Regulation of HSF1-mediated heat shock response
(6) Interferon signaling
(7) Transport of ribonucleoproteins into the host nucleus
(8) Non-coding RNA metabolism
(9) Interactions of VPR with host cellular proteins
(10) Sumoylation of RNA binding proteins
(11) Transport of mature mRNA derived from an intronless transcript
(12) Sumoylation of DNA replication proteins
(13) Nuclear envelope breakdown
(14) SNRNP assembly
(15) Cellular response to heat stress
(16) Nuclear pore complex (NPC) disassembly
(17) ISG15 antiviral mechanism
(18) Sumo E3 ligases sumoylate target proteins
(19) Export of viral ribonucleoproteins from the nucleus
(20) REV-mediated nuclear export of HIV RNA
(21) Transport of mature mRNAs derived from intronless transcripts
(22) NEP NS2 interacts with the cellular export machinery
(23) Sumoylation of DNA damage response and repair proteins
(24) tRNA processing
(25) Interactions of REV with host cellular proteins
(26) Transport of the SLBP independent mature mRNA
(27) Antiviral mechanism by IFN-stimulated genes
(28) Viral messenger RNA synthesis
(29) Glucose transport
(30) Hexose uptake
(31) Sumoylation
(32) NS1 mediated effects on host pathways
(33) Host interactions with influenza factors
(34) Transport of the SLBP dependant mature mRNA
(35) Signaling by FGFR2
(36) Signaling by FGFR
(37) tRNA processing in the nucleus
(38) Late phase of HIV life cycle

Figure S3. Heat map showing the leading edge subsets (LES) of pathways in the 'nucleocytoplasmic transport' cluster. The heat map fill color indicates the pairwise comparison/s in which each LES protein was implicated.

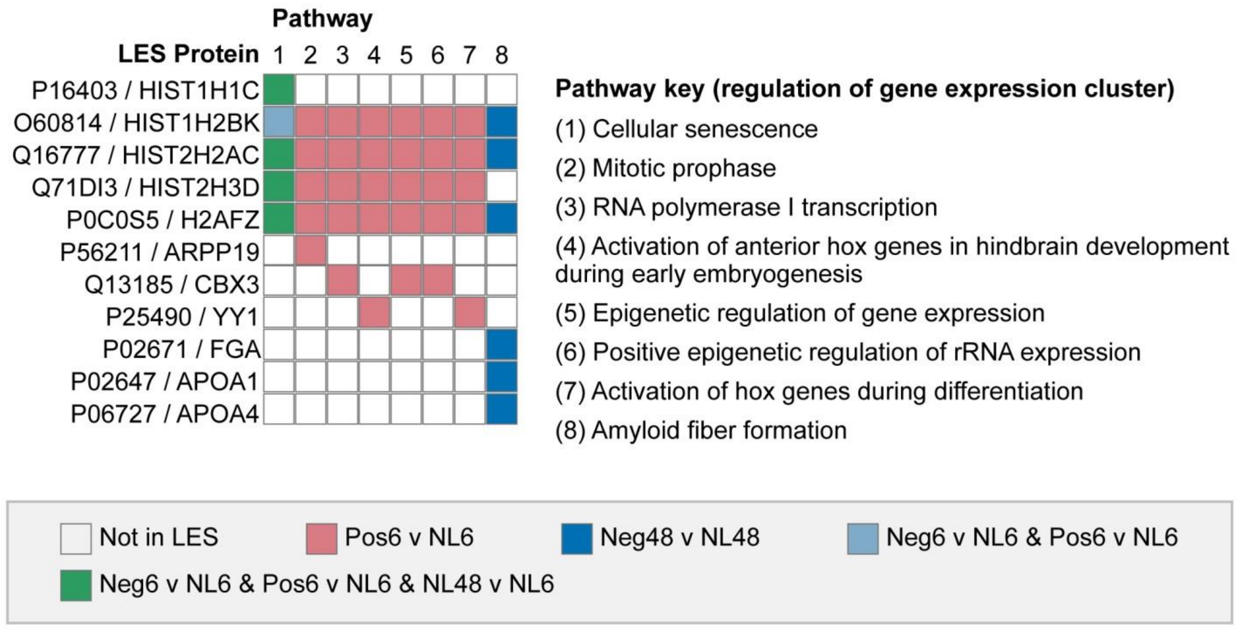

Figure S4. Heat map showing the leading edge subsets (LES) of pathways in the 'regulation of gene expression' cluster. The heat map fill color indicates the pairwise comparison/s in which each LES protein was implicated.

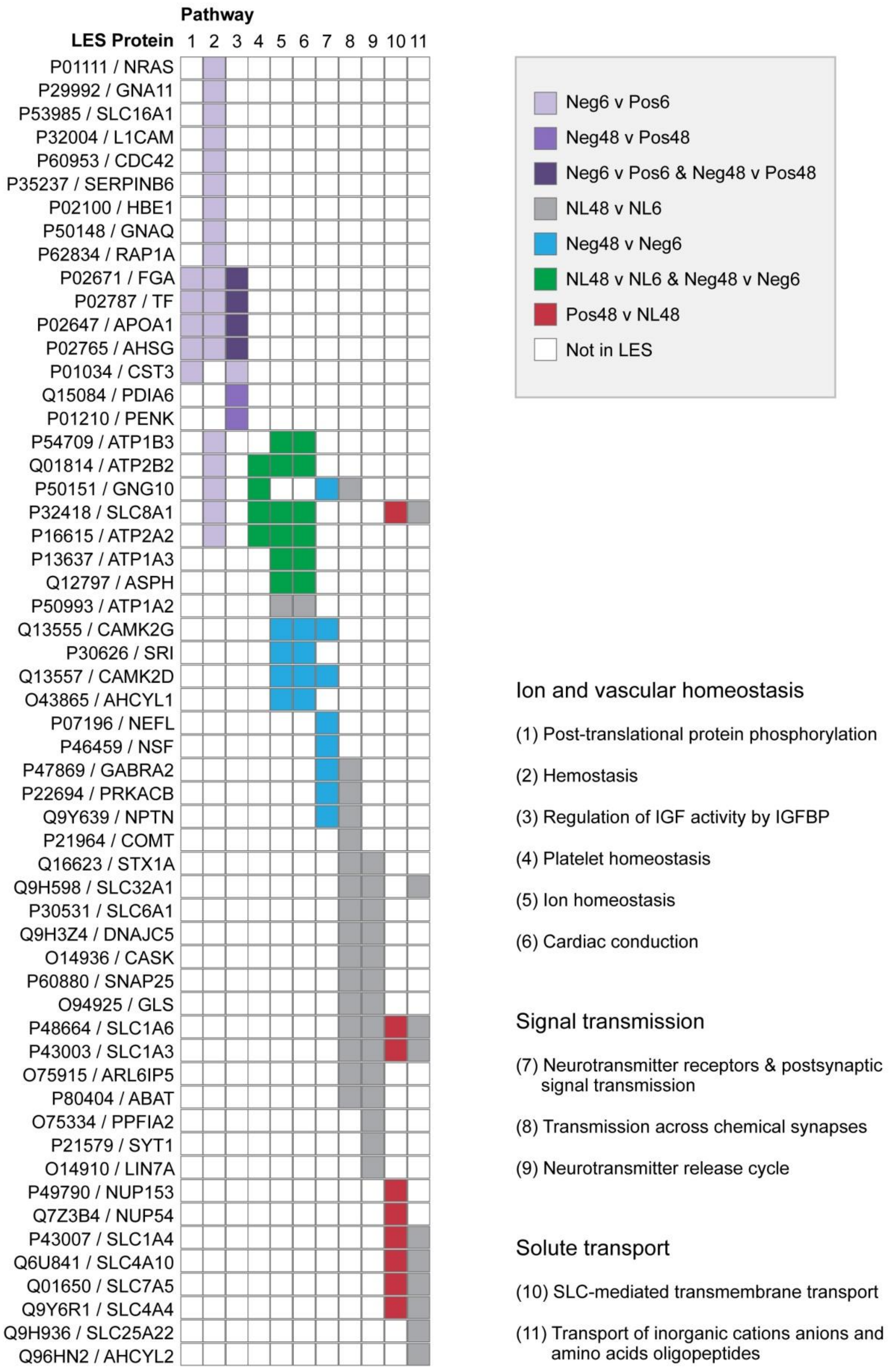

Figure S5. Heat map showing the leading edge subsets (LES) of pathways in the 'ion and vascular homeostasis', 'signal transmission', and 'solute transport' clusters. The heat map fill color indicates the pairwise comparison/s in which each LES protein was implicated.
